# Renal function affects absorbed dose to the kidneys and haematological toxicity during ^177^Lu-DOTATATE treatment

**DOI:** 10.1007/s00259-015-3001-1

**Published:** 2015-02-06

**Authors:** Johanna Svensson, Gertrud Berg, Bo Wängberg, Maria Larsson, Eva Forssell-Aronsson, Peter Bernhardt

**Affiliations:** 1 Department of Oncology, Sahlgrenska University Hospital, SE 413 45 Göteborg, Sweden; 2 Department of Surgery, Sahlgrenska University Hospital, Göteborg, Sweden; 3 Department of Radiation Physics, Institute of Clinical Sciences, The Sahlgrenska Academy, University of Gothenburg, Göteborg, Sweden; 4 Department of Medical Physics and Medical Bioengineering, Sahlgrenska University Hospital, Göteborg, Sweden

**Keywords:** ^177^Lu-DOTATATE therapy, PRRT, Neuroendocrine tumours, Dosimetry

## Abstract

**Purpose:**

Peptide receptor radionuclide therapy (PRRT) has become an important treatment option in the management of advanced neuroendocrine tumours. Long-lasting responses are reported for a majority of treated patients, with good tolerability and a favourable impact on quality of life. The treatment is usually limited by the cumulative absorbed dose to the kidneys, where the radiopharmaceutical is reabsorbed and retained, or by evident haematological toxicity. The aim of this study was to evaluate how renal function affects (1) absorbed dose to the kidneys, and (2) the development of haematological toxicity during PRRT treatment.

**Methods:**

The study included 51 patients with an advanced neuroendocrine tumour who received ^177^Lu-DOTATATE treatment during 2006 – 2011 at Sahlgrenska University Hospital in Gothenburg. An average activity of 7.5 GBq (3.5 – 8.2 GBq) was given at intervals of 6 – 8 weeks on one to five occasions. Patient baseline characteristics according to renal and bone marrow function, tumour burden and medical history including prior treatment were recorded. Renal and bone marrow function were then monitored during treatment. Renal dosimetry was performed according to the conjugate view method, and the residence time for the radiopharmaceutical in the whole body was calculated.

**Results:**

A significant correlation between inferior renal function before treatment and higher received renal absorbed dose per administered activity was found (*p* < 0.01). Patients with inferior renal function also experienced a higher grade of haematological toxicity during treatment (*p* = 0.01). The residence time of ^177^Lu in the whole body (range 0.89 – 3.0 days) was correlated with grade of haematological toxicity (*p* = 0.04) but not with renal absorbed dose (*p* = 0.53).

**Conclusion:**

Patients with inferior renal function were exposed to higher renal absorbed dose per administered activity and developed a higher grade of haematological toxicity during ^177^Lu-DOTATATE treatment. The study confirms the tolerability of PRRT in patients with an advanced neuroendocrine tumour but indicates that patients with inferior renal function are at risk of being exposed to higher absorbed doses to normal tissue on treatment.

## Introduction

Peptide receptor radionuclide therapy (PRRT) using somatostatin analogues has now been a valuable treatment option for advanced neuroendocrine tumours for more than 10 years. Kwekkeboom et al. in Rotterdam were the first to report the effectiveness and safety of this treatment [1] and the same group reported a positive impact on quality of life [2]. PRRT is well tolerated in general but normal tissue is exposed to radiation to different degrees. The dose-limiting organ is usually the kidney, because of active reabsorption of the radionuclide-labelled somatostatin analogue. Renal uptake is mediated by the endocytic receptor megalin in the proximal tubuli [3]. The amount of PRRT given has often been restricted by an absorbed dose limit of 23 Gy to the kidneys on the basis of earlier established knowledge about renal absorbed dose tolerance from external beam radiation [4].

Efforts have been made to find tolerance doses for the kidneys that better match the risks from radionuclide therapy with its different dose rates, dose distributions and fractionation characteristics compared to external beam radiation [5–7]. Bodei et al. calculated the biological effective dose (BED) using a linear quadratic model adapted to radionuclide therapy. They showed that a safe renal absorbed dose limit for PRRT (^90^Y-DOTATOC and ^177^Lu-DOTATATE) might be a BED of around 40 Gy in patients without risk factors for renal toxicity and 28 Gy in patients with certain risk factors, including hypertension, diabetes mellitus and other conditions known to affect renal function [8]. Also Gupta et al. recently demonstrated the relevance of using different absorbed dose limits to the kidneys based on renal function. In their study, PRRT showed a greater effect on renal function in patients with inferior glomerular filtration rate (GFR) initially than in patients with superior renal function [9].

In clinical practice haematological toxicity sometimes becomes relevant; a toxicity of grade 3 or 4 according to the Common Terminology Criteria for Adverse Events (CTCAE) of the US National Cancer Institute has been reported in approximately 10% of patients after one or more PRRT treatments of [9–12]. Possible risk factors for haematological toxicity include previous chemotherapy, low creatinine clearance and bone metastases [13]. Other toxicities such as liver toxicity might occur during PRRT but much less frequently [10]. The normal tissue reported to receive the highest absorbed dose is the spleen [14, 15]. However, compromised spleen function has not been reported after PRRT.

It has been reported that transient deterioration of renal function can have an important effect on the absorbed dose to normal tissue from PRRT [16]. The aim of this study was to analyse what impact renal function has on renal absorbed dose and the development of haematological toxicity. Factors known to affect renal and bone marrow function including hypertension, diabetes, older age, previous chemotherapy and comedication were noted [8, 17]. Because tumour burden and whole-body content kinetics for the radiopharmaceutical could affect the radiation exposure of kidneys and bone marrow, these factors were also analysed.

## Materials and methods

### Patients and treatment characteristics

This retrospective study was approved by the Regional Ethics Review Board in Gothenburg and performed in accordance with the principles of the Declaration of Helsinki and national regulations. The need for written informed consent was waived. From March 2006 to December 2011, 51 patients with an advanced neuroendocrine tumour were treated with ^177^Lu-DOTATATE at Sahlgrenska University Hospital in Gothenburg (Table 1). Patients considered for treatment had progressive disease detected by radiology, enhanced carcinoid symptoms or increasing tumour markers. All patients had a WHO performance status of 0 – 2 and a tumour uptake on somatostatin receptor scintigraphy (^111^In-DTPA^0^ octreotide, Octreoscan®; Mallinckrodt) judged to exceed normal liver uptake. Patients with moderately reduced renal function were accepted, that is those with a GFR of 40 ml/min (i.e. chronic kidney disease stage 3 [18]) or more, based on ^51^Cr-EDTA clearance. An average amount of 7.5 GBq (3.5 – 8.2 GBq) ^177^Lu-DOTATATE was given as a 30-min intravenous infusion coadministered with kidney-protective amino acids (2.5 % lysine and 2.5 % arginine in 1 L of 0.9 % NaCl; rate of infusion 250 mL/h) on three (one to five) occasions 8 weeks (6 – 10 weeks) apart. The number of treatments was usually limited by the absorbed dose to the kidneys, but in some patients the number of treatments was limited by disease progression (three patients) or persistent haematological toxicity (four patients).Subgroups (26 and 33) of these patients had previously been studied concerning safety, efficacy and variations in renal dosimetry [19, 20].

**Table 1:** Patient characteristics

Characteristic	No. of patients
Gender	
Male	31
Female	20
Primary tumour origin	
Small intestine	30
Pancreas	11
Rectum	4
Other	6
Metastatic organs	
Liver	36
Lymph nodes/soft tissue	33
Bone	18
Other	6
Previous treatment	
Hepatic artery embolization	16
Chemotherapy	15
External beam radiation	3
Liver transplantation	4

### Dosimetry

For all analyses in this study, the absorbed dose to the kidneys and the whole-body residence time for ^177^Lu for the first treatment cycle were used. The conjugate view method [21] was applied to planar images obtained 1.5, 24, 48 and 168 h after injection. The gamma cameras used were a Picker IRIX (Marconi, Philips, The Netherlands) and a Millennium VG Hawkeye (General Electric Medical Systems, Milwaukee, WI), equipped with medium-energy parallel-hole collimators. The CT images were generated by the Millennium VG Hawkeye system.

The effective attenuation coefficient and sensitivity for the gamma cameras were determined by planar scintigraphy of a planar ^177^Lu source equal to the cross-sectional area of a standard kidney placed at different depths in a phantom of tissue equivalent material. The counts in a region of interest (ROI) drawn around the ^177^Lu source were recorded from the planar images. A monoexponential curve was fitted to the number of counts versus depth data, and the effective attenuation coefficient and sensitivity were determined. The thickness of the body over the kidney and the kidney thickness were determined from a low-resolution CT scan performed 24 h after injection. The counts in the anterior and posterior images in a ROI around the kidney were recorded and the counts in a background ROI located caudal to the kidney ROI were subtracted. The background counts were adjusted to account for the overlying and underlying tissue in the kidney ROI. In patients in whom it was not possible to separate the liver uptake from the right kidney uptake, or to separate tumour uptake from the uptake in one of the kidneys, the activity in that kidney was calculated from only a part of the kidney (right kidney in 33 patients, left kidney in 33 patients). If the kidney could not be delineated at all (5 patients), the activity concentration was determined in a SPECT image 24 h after injection and the kinetics from the left kidney were used.

To calculate the absorbed dose to the kidneys, the effective attenuation coefficient, sensitivity, anterior and posterior counts, and patient and kidney thickness were inserted into the conjugate view formula. This procedure is described elsewhere [20]. The activity obtained was divided by the kidney mass, which was estimated assuming an ellipsoid, and the lengths of the three axes were determined from high-resolution CT images obtained before treatment. A monoexponential curve fit was applied to the time–activity data and the accumulated specific activity calculated. The absorbed dose was estimated by assuming local absorption of the charged particles emitted from ^177^Lu (mean energy per decay equal to 147 keV [22]).

To evaluate other factors possibly affecting radiation exposure of normal tissue, the variation in residence time of the radiopharmaceutical in the whole body was analysed. The whole-body content was determined by the conjugate view method, and in the 24 patients who had not urinated this resulted in a calculated whole-body activity from the planar image obtained 1.5 h after injection that was 94 ± 8.5 % of the injected activity using the patient thickness over the kidneys. There was a trend towards an increase in estimated activity with increasing patient thickness (1.1 %/cm). The patient thickness in the study was between 16 and 28 cm making a maximal deviation of 13 %. The patient thickness in the conjugate view formula was therefore calibrated to make the measured activity at 1.5 h after injection equal to the injected activity of the patients who had not urinated, resulting in a relative standard deviation of 8 % with no patient thickness dependence. This method for calibrating patient thickness was applied in all 51 patients to determine the whole-body residence time.

Bone marrow dosimetry was not performed in this study because previous studies had shown that the absorbed dose to the bone marrow is usually low [15, 23]. This has recently been confirmed by a study of bone marrow dosimetry during ^177^Lu-DOTATATE therapy indicating that the absorbed dose to the bone marrow is less than 0.2 Gy per treatment cycle of 7.4 GBq [24].

### Tumour burden

**Fig. 1:**
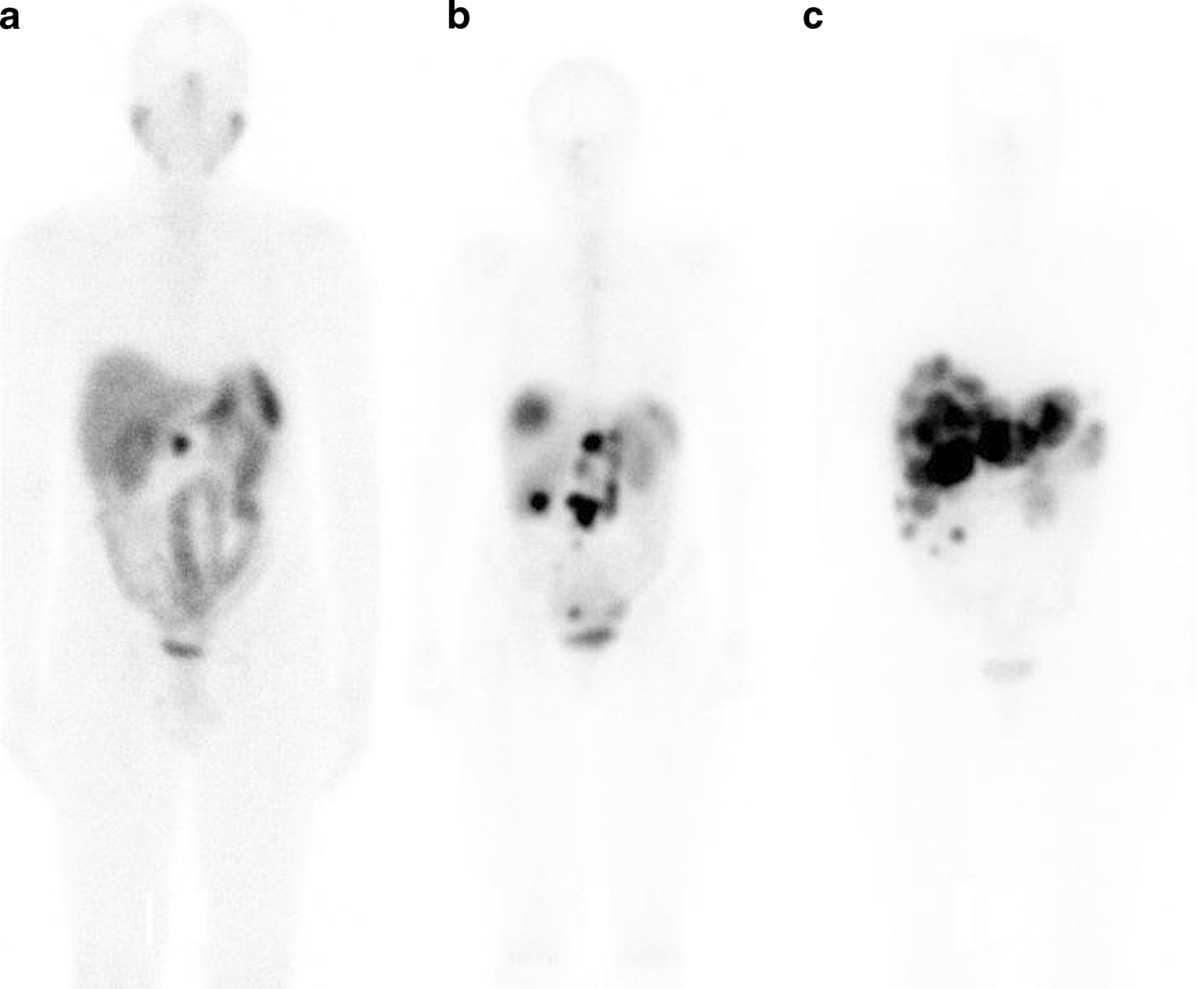
Examples of patients classified as having a small (**a** grade 1), medium (**b** grade 3) and large (**c** grade 5) tumour burden. The patient with a small tumour burden (**a**) has a single tumour located in the abdomen, the patient with a medium tumour burden (**b**) has multiple tumours in the abdomen, and the patient with a large tumour burden (**c**) has multiple tumours in the liver involving around 50 % of the parenchyma

Patient tumour burden was visually estimated in the planar images obtained 24 h after injection from the first treatment and graded from 1 (minor tumour burden) to 5 (major tumour burden; Fig. 1). For comparative analysis patients graded 1 or 2 were considered to have a small tumour burden, patients graded 3 were considered to have a medium tumour burden and patients graded 4 or 5 were considered to have a large tumour burden.

### Renal and haematological toxicity

The patients were monitored clinically and according to renal and haematological toxicity during the treatment period by regular measurements of serum creatinine and haemoglobin, leucocytes and platelets. Toxicity was graded from 0 to 5 according to the CTCAE 4.0 of the NCI.

### Statistical analysis

To report normally distributed continuous variables, means and standard deviation are used. Possible relationships between variables were evaluated by independent-sample *t* tests and linear regression. For all statistical analyses the statistical software IBM SPSS 19 was used and *p* values less than 0.05 were considered significant.

## Results

**Fig. 2:**
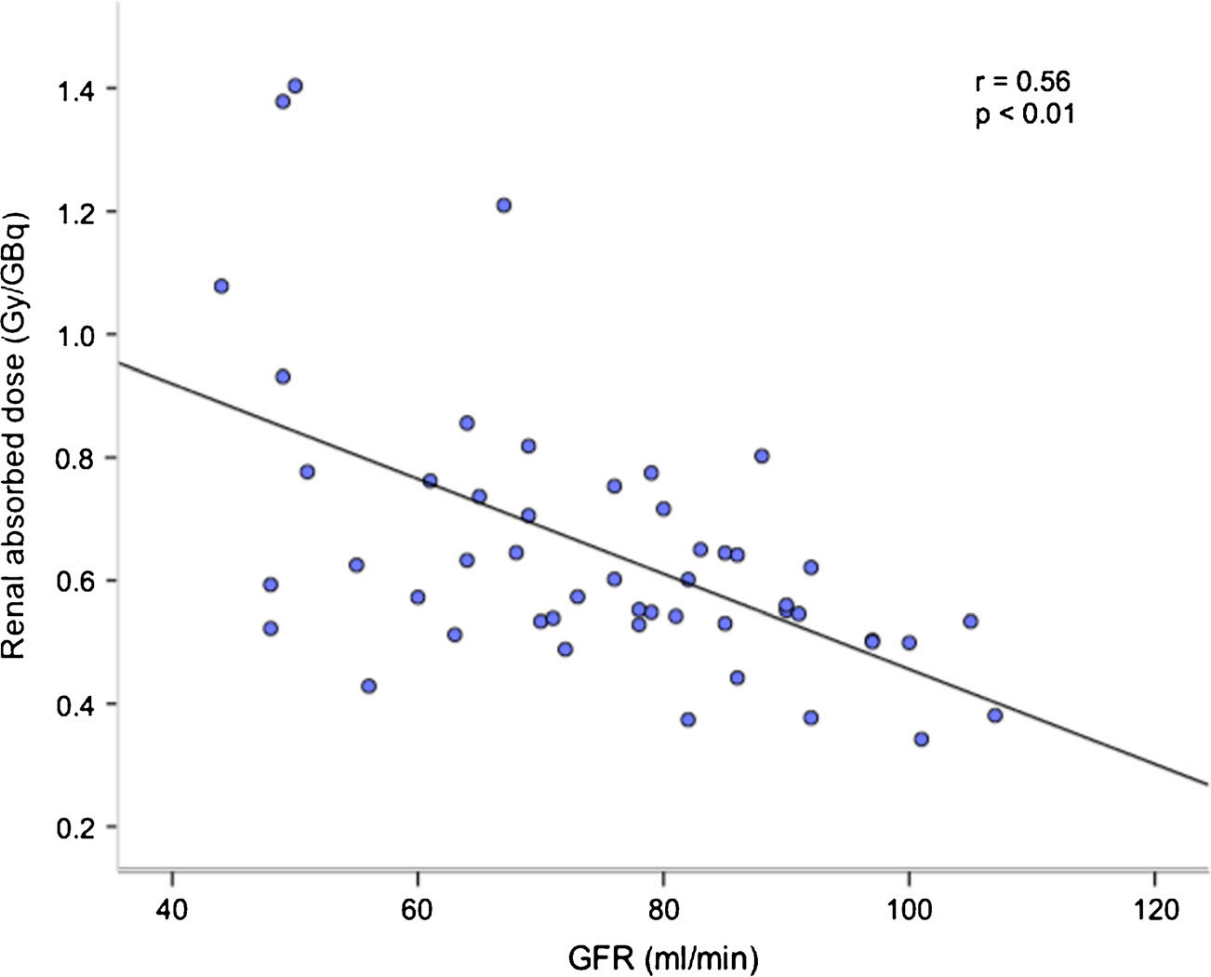
Patients with inferior renal function estimated by GFR (from ^51^Cr-EDTA clearance) received higher renal absorbed doses per injected amount of ^177^Lu-DOTATATE

Patients with inferior renal function, estimated by GFR before treatment, were exposed to significantly higher renal absorbed doses (*p* < 0.01, Fig. 2) during their first ^177^Lu-DOTATATE treatment.. The data showed considerable variation and more markedly so in patients within the lower range of GFR. Patients with GFR ≤60 ml/min (i.e. chronic kidney disease stage 1 [18]; 10 patients) received renal absorbed doses of 0.83 ± 0.35 Gy/GBq and patients with GFR ≥90 ml/min (11 patients) received renal absorbed doses of 0.49 ± 0.09 Gy/GBq (*p* < 0.01). The other patients (GFR >60 to <90 ml/min) received renal absorbed doses of 0.64 ± 0.16 Gy/GBq.

Conditions known to affect renal function, including age, comorbidity (hypertension or diabetes mellitus), prior chemotherapy and comedication, were explored to determine if there were differences in received absorbed dose to the kidneys. Renal function declines physiologically with age, and a trend toward this was seen among patients in this study, but the differences were not statistically significant. Baseline GFR was 65 ± 16 ml/min in patients older than 70 years (8 patients) and 77 ± 16 ml/min in younger patients (43 patients, *p* = 0.07), and consequently patients older than 70 years at the time of treatment were not exposed to significantly higher renal absorbed doses (0.76 ± 0.31 Gy/GBq compared with 0.62 ± 0.20 Gy/GBq for the younger group, *p* = 0.12). Patients with hypertension (15 patients) and patients previously exposed to chemotherapy (15 patients) did not differ in their renal function as estimated by GFR compared with the other patients. GFR was 76 ± 16 ml/min in patients without hypertension and 73 ± 17 ml/min in patients with hypertension (*p* = 0.56), and baseline GFR was 73 ± 16 ml/min in patients not exposed to chemotherapy and 79 ± 17 ml/min in those who had been exposed to chemotherapy (*p* = 0.26). Renal absorbed doses were similar in these groups. Renal absorbed dose/injected activity was 0.63 ± 0.20 Gy/GBq in patients without hypertension and 0.69 ± 0.28 Gy/GBq in patients with hypertension (*p* = 0.39). Renal absorbed dose was 0.68 ± 0.23 Gy/GBq in patients not exposed to chemotherapy and 0.57 ± 0.21 Gy/GBq in those who had been exposed to chemotherapy (*p* = 0.12). Patients with diabetes mellitus (two patients) and patients on medication with a potential effect on renal function, such as nonsteroidal antiinflammatory drugs (two patients), were too few to analyse. All patients were monitored according to their renal function during the treatment period, with no signs of renal toxicity detected, as estimated using serum creatinine.

**Fig. 3:**
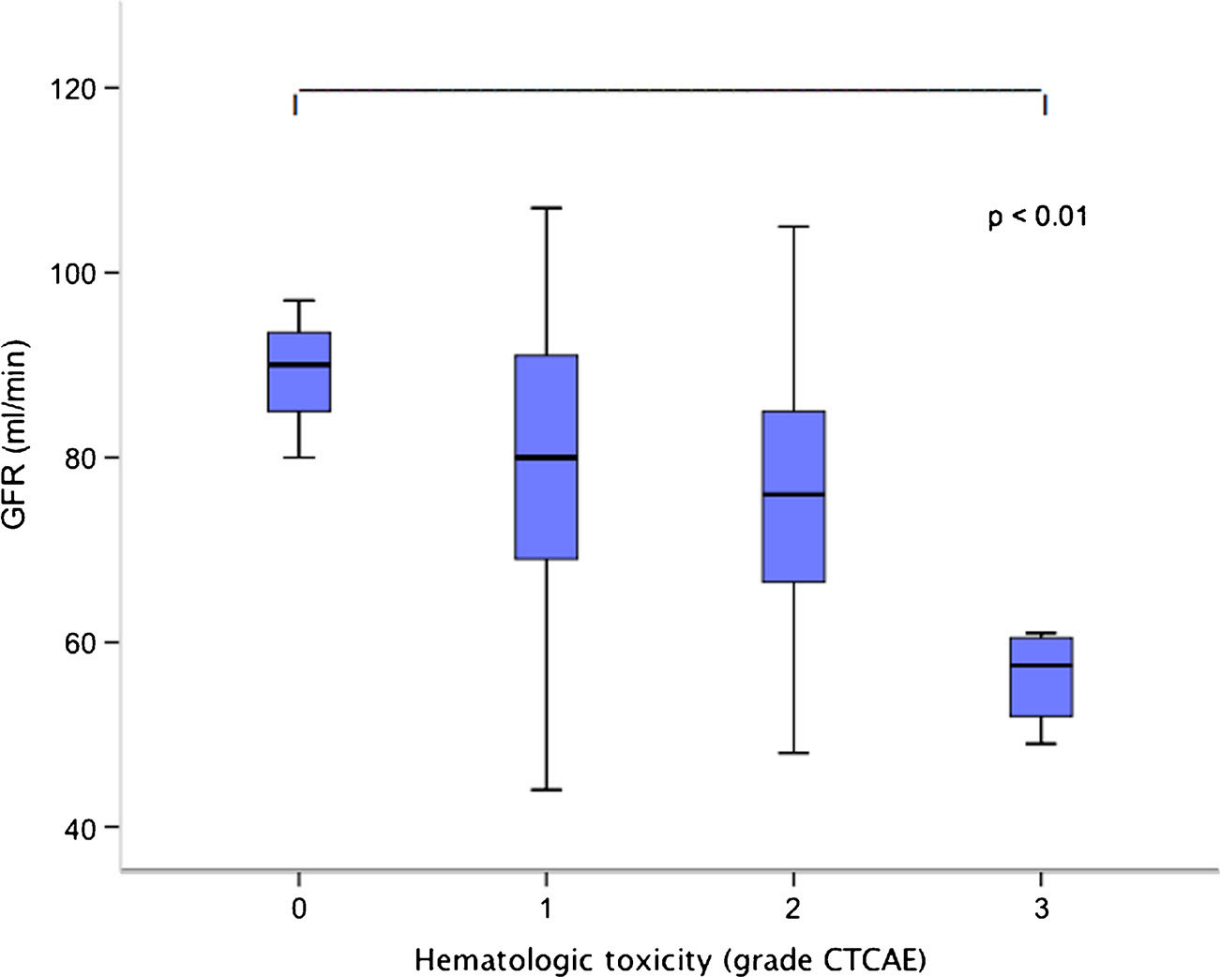
Patients with inferior renal function tended to develop a higher grade of haematological toxicity during ^177^Lu-DOTATATE treatment according to CTCAE

Renal function was also correlated with the development of haematological toxicity during treatment. GFR was 89 ± 9 ml/min in patients who did not experience haematological toxicity (grade 0 according to CTCAE) and 56 ± 6 ml/min in those developing the highest toxicity (grade 3). Although the groups were small (three patients with CTCAE grade 0, four patients with grade 3), the mean values differed significantly (*p* < 0.01, Fig. 3). Patients with bone metastases could be at higher risk of developing haematological toxicity. In this study 11 of 18 patients (61 %) with bone metastases developed grade 2 or 3 haematological toxicity compared with 13 of 30 (43 %) of the other patients. Previous exposure to chemotherapy may also affect bone marrow function, but it was not associated with a higher frequency of haematological toxicity in this study. Of 34 patients not exposed to chemotherapy, 16 (47 %) experienced grade 2 or 3 haematological toxicity, compared with 7 of 14 patients previously exposed to chemotherapy (50 %). No patients developed grade 4 or 5 haematological toxicity.

**Fig. 4:**
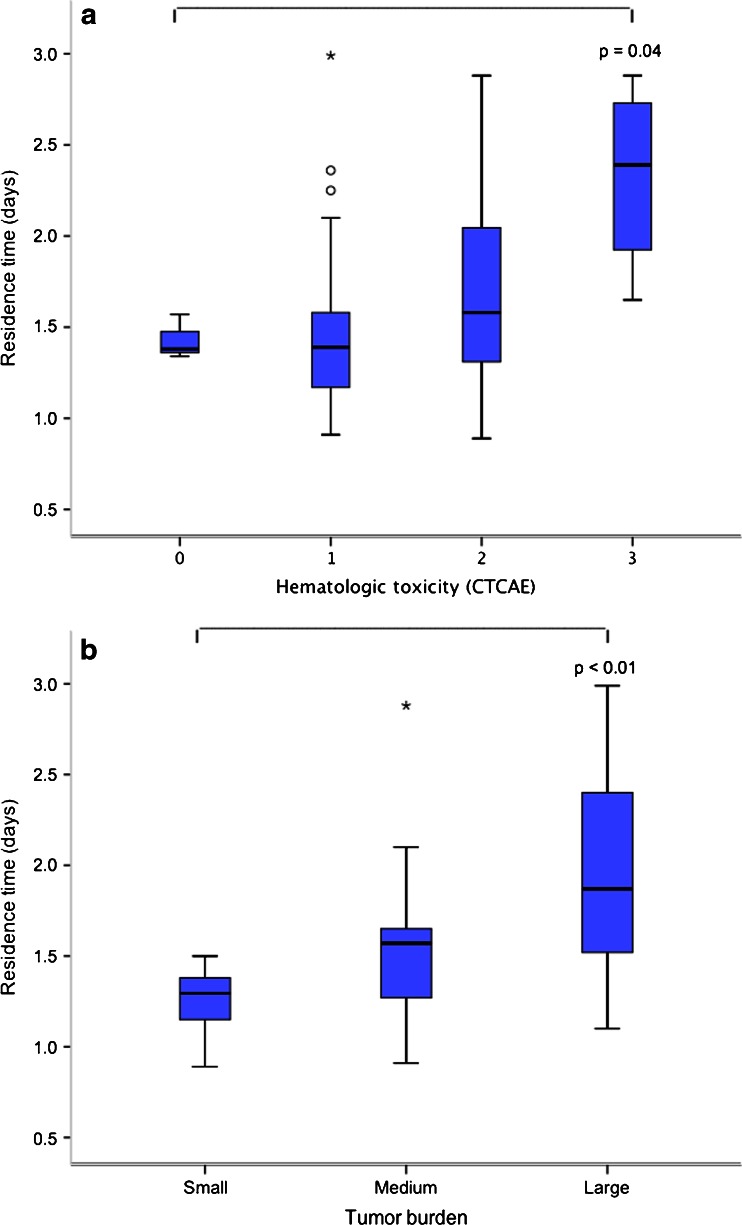
Longer whole-body residence time of ^177^Lu was associated with a higher grade of haematological toxicity (**a**) and a larger tumour burden (**b**)

To evaluate other factors possibly affecting radiation exposure of normal tissue during PRRT treatment, variations in residence time for the radiopharmaceutical in the whole body as well as tumour burden were analysed. There was no correlation between renal function and the whole-body residence time of ^177^Lu, and the residence time did not affect renal absorbed dose. However, patients with a longer whole-body residence time of ^177^Lu tended to develop a higher grade of haematological toxicity. Residence time was 2.3 ± 0.5 days in patients who developed grade 3 haematological toxicity and 1.4 ± 0.1 days in patients who did not experience haematological toxicity (*p* = 0.04, Fig. 4a).

To evaluate the relationship between tumour burden and radiation exposure of normal tissue, tumour burden was visually estimated as small (grade 1 and 2), medium (grade 3) or large (grade 4 and 5) in planar images obtained 24 h after treatment. Residence time was 2.0 ± 0.7 days in patients with a large tumour burden and 1.3 ± 0.2 days in patients with a small tumour burden (*p* < 0.01, Fig. 4b). GFR was 72 ± 14 ml/min in 23 patients with a large tumour burden and 84 ± 11 ml/min in 11 patients with a small tumour burden (*p* = 0.02). Despite differences in renal function, renal absorbed dose per injected activity of ^177^Lu-DOTATATE did not differ between patients with a large and a small tumour burden: 0.63 ± 0.20 Gy/GBq vs. 0.62 ± 0.11 Gy/GBq (*p* = 0.88). However, the frequency of haematological toxicity seemed higher among patients with a large tumour burden: among patients in whom haematological toxicity could be evaluated, 12 of 21 (57 %) with a large tumour burden and 4 of 11 (36 %) with a small tumour burden experienced grade 2/3 haematological toxicity.

## Discussion

Patients with inferior renal function as estimated by GFR were exposed to significantly higher renal absorbed dose during their first ^177^Lu-DOTATATE treatment in this study. The variation was considerable, and appeared to be higher among patients with inferior renal function. This variation is probably due to both physical and biological issues. Physical variations are known to be larger in the two-dimensional conjugate view method used, compared to three-dimensional dosimetry [20, 25]. A correlation was found between GFR and the two-dimensional estimated renal absorbed dose in this study, but a patient-specific prediction of the renal absorbed dose from the GFR was not possible due to the substantial variation. Future studies with three-dimensional renal dosimetry could improve the precision and enable a patient-specific use of the correlation found. A disadvantage of three-dimensional dosimetry is that only a limited part of the body is usually studied, which is insufficient for whole-body dosimetry. Nevertheless, the physical variations would be expected to remain constant independent of renal function. Therefore, the observed increased variation seen along with decreasing renal function may be due to biological phenomena. One reason for this could be heterogeneity in the kind of renal impairment present among the patients. The method chosen to estimate renal function in this study, GFR, is a measure of glomerular function and does not measure tubular function. Patients with lower GFR might differ in tubular function more markedly, which could affect tubular reabsorption ability and exposure to the radiopharmaceutical from the primary urine. Tubular function was not estimated in this study, for example by measuring the ratio of protein HC (α1-microglobulin) to creatinine in urine [26] or by performing a ^99m^Tc-MAG_3_ renal scan [27].

Other factors known to affect the kidneys are hypertension and previous chemotherapy. Guerriero et al. [28] found a significant difference in renal absorbed dose between patients with hypertension and previous chemotherapy and other patients. In our study neither hypertension nor previous chemotherapy seemed to affect the absorbed dose to the kidneys. The reason for these different findings could be that patients in our study had better renal function at the time of treatment. Patients with newly diagnosed, well-regulated hypertension often have normal renal function while patients with longstanding, insufficiently regulated hypertension may have developed clinically significant renal dysfunction [29]. Patients with previous chemotherapy are also a heterogeneous group in whom treatment dosages vary, as do agents. Of the 15 patients in this study who had received chemotherapy, the therapy given to 13 was known to potentially affect the kidneys (streptozotocin or cisplatin) [30], but the GFR did not differ between these patients and the other patients at the time of PRRT. The time between completed chemotherapy and the start of PRRT, a median of 9 months in this study, will probably also affect the condition of the kidneys.

In addition to being exposed to higher renal absorbed doses, patients with inferior renal function also tended to experience a higher grade of haematological toxicity according to CTCAE. Another factor that can potentially affect the bone marrow and the consequent development of haematological toxicity is the presence of bone metastases. These patients did experience a higher frequency of moderate haematological toxicity (grade 2/3) in this study, as previously shown by Kam et al. [13]. However, no correlation was seen between previous exposure to chemotherapy and haematological toxicity, in contrast to the findings of Kam et al. [13]. As mentioned above, this may be because of heterogeneity in the chemotherapy group. It should also be taken into account that the subgroups that were compared, developing grade 0 and grade 3 haematological toxicity, respectively, were small.

To evaluate other differences in radiation exposure of normal tissue, the residence time of ^177^Lu in the whole body as well as patient tumour burden were analysed. It might have been expected that the whole-body residence time would increase with a lower GFR, but this was not seen in the present study. We are aware of the uncertainties in the method used, but the lack of correlation still indicates that factors other than renal function probably contribute to the residence time. One factor affecting the residence time of ^177^Lu in this study was tumour burden. Interestingly, a longer residence time was associated with an increase in haematological toxicity but not renal absorbed dose. One reason for this may be that a higher tumour burden increases the radiation exposure of the bone marrow whereas the kidneys may instead be protected from irradiation because a higher proportion of the radiopharmaceutical accumulates in the tumour. Garske et al. found that decreasing tumour burden during multiple ^177^Lu-DOTATATE treatments was associated with a higher renal absorbed dose but a lower estimated bone marrow absorbed dose [31, 32]. The estimated renal absorbed dose did not differ between patients with a large and a small tumour burden in this study. A reason for this could have been that patients with a smaller tumour burden had a better estimated renal function before treatment, which could have compensated for the expected tendency to receive a relatively higher renal absorbed dose, as noted by Garske et al. [31].

More detailed bone marrow dosimetry studies [33, 34] have shown no evident correlation between received bone marrow absorbed dose and the development of haematological toxicity. This indicates that other factors affect haematological toxicity. Sabet et al. found that during the long-term follow-up of PRRT patients, a history of splenectomy (in 16 patients) was inversely associated with the incidence of haematological toxicity [11]. The spleen, where blood cells are pooled, is known to be exposed to the highest absorbed doses during PRRT [14]. However, a possible correlation between the absorbed dose to the spleen and haematological toxicity remains to be studied.
